# Metal-Free A_2_/B_2_-Type Azide–Alkyne Polyaddition: Effect of Azides Structure on Their Reactivity and Properties of Polymerization Products

**DOI:** 10.3390/polym17141909

**Published:** 2025-07-10

**Authors:** Andrey Galukhin, Roman Aleshin, Alexander Gerasimov, Alexander Klimovitskii, Roman Nosov, Liana Zubaidullina, Sergey Vyazovkin

**Affiliations:** 1 Alexander Butlerov Institute of Chemistry, Kazan Federal University, 18 Kremlevskaya Street, 420008 Kazan, Russia; msizhir-kniga@mail.ru (R.A.); alexander.gerasimov@kpfu.ru (A.G.); aklimovi@mail.ru (A.K.); romanosow@mail.ru (R.N.); lian-usman@mail.ru (L.Z.); 2 Department of Chemistry, University of Alabama at Birmingham, 901 S. 14th Street, Birmingham, AL 35294, USA

**Keywords:** azide–alkyne polyaddition, 1,3-dipolar cycloaddition, reactivity, kinetics, thermal analysis, isoconversional approach

## Abstract

Non-isothermal calorimetry is performed to study the kinetics of metal-free A_2_/B_2_-type azide–alkyne polyaddition between the dipropargyl ether of bisphenol A with different organic diazides. The diazide structure is varied to probe the effect of the nature of a hydrocarbon spacer between the azide groups on their reactivity. Isoconversional analysis demonstrates that the polymerization processes are characterized by the same activation energy of 84 kJ mol^−1^ for all studied diazides. It is found that diazides with aromatic spacers demonstrate ~1.6 times higher reactivity than that of diazides with the alkyl spacer. The difference in the reactivity is explained by the difference in the electronic effects of the hydrocarbon spacers on the azide groups as well as by the difference in their steric availability. The veracity of the obtained kinetic parameters is validated by a polymerization test at the time–temperature conditions predicted from the obtained kinetic data followed by independent assessment of the monomer conversion using FTIR.

## 1. Introduction

Designing functional polymers is a vigorously evolving trend in polymer chemistry. It focuses on creating polymers with specific properties and functions tailored to particular applications as opposed to traditional polymers whose usage is typically generic [1]. Poly(1,2,3-triazole)s possess rich functionality that affords a variety of diverse applications. The presence of 1,2,3-triazolic fragments in polymer chains enhances adhesive [2,3,4] and anticorrosion characteristics [5,6], increases flame retardation performance [7], and promotes self-healing [8,9,10] and shape memory properties [4,11]. Cross-linking of poly(1,2,3-triazole)s by alkylated agents (e.g., α,ω-dihalogenalkanes) yields poly(1,2,3-triazolium) salts with ion-conducting and vitrimeric properties [12,13,14]. A significant advantage of poly(1,2,3-triazole)s is that they can be readily synthesized by azide–alkyne polyaddition (AAP).

There exist two types of monomers used for AAP. Monomers of the first type contain both azide and alkyne functions in the same molecule that permits performing a single-component reaction. The ratio of azide (A) to alkyne (B) groups combined in one molecule, i.e., AB, A_2_B, AB_2,_ etc., determines the topology of macromolecules and allows one to synthesize linear [15,16,17,18] or branched [19] polymers. The main advantage of these monomers is that they set the exact stoichiometry of reacting groups at the molecular level, which in theory makes it possible to achieve a high degree of polymerization [18]. The second type of monomers contain azide and alkyne groups separately in different molecules [20,21,22], which assumes participation of at least two different reactants in AAP. Monomers of the second type can be more readily synthesized and stored compared to those of the first type, which makes them more convenient to handle.

Control over the polymerization rate of AAP is crucial for designing self-healing poly(1,2,3-triazole)s as a factor for the production of materials with the desired repair conditions [10]. Moreover, a controllable heat production rate of AAP allows conducting the so-called “out-of-autoclave” polymerization [23]. This technology is highly sought-after for the preparation and repair of large composite panels used in construction applications without the utilization of expensive autoclaves [24]. The most common approach for the acceleration of AAP is the use of transition metal-based catalysts (e.g., Cu(I) [21,22,25], Ru(II) [26,27], Ni(II) [28], etc.), which significantly increase the reaction rate and secure markedly milder polymerization conditions. However, the application of metal-based catalysts inevitably contaminates the resulting polymer with respective metals that diminishes its oxidative and thermal stabilities [29].

**Figure 1:**
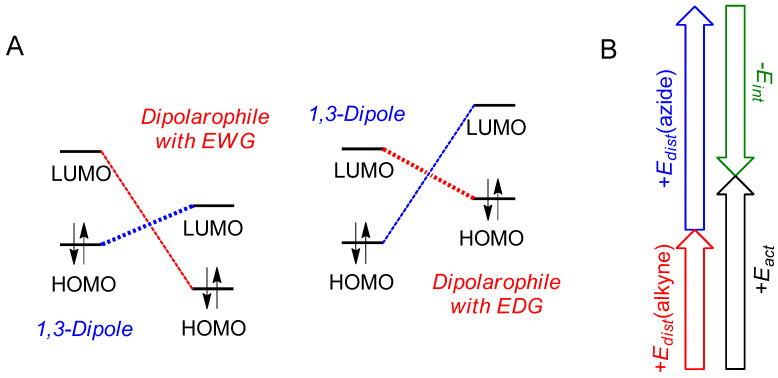
Scheme of HOMO-LUMO interactions in 1,3-dipolar cycloaddition. Preferred paths are shown in bold (**A**). Schematic contributions of distortion and interaction energies of reactants into activation energy of reaction (**B**).

The above problem motivates one to search for solutions to controlling the AAP kinetics, which do not involve metal catalysts [20,30]. One possible approach to addressing this problem is to control the reactivity of the monomers by controlling their structure. Although quantitative structure–reactivity studies have never been performed for AAP, in general, it is known that 1,3-dipolar cycloaddition reactions, of which AAP is a special case, are characterized by the U-shaped dependence of the rate constant on the electron density of the multiple bonds of dipolarophiles (alkenes, alkynes, etc.) [31,32,33]. It means that both electron-withdrawing (EWG) and electron-donating (EDG) groups attached to dipolarophiles can accelerate the kinetics of the reaction. Such dependence is explained qualitatively by the frontier molecular orbital (FMO) theory [34], according to which 1,3-dipolar cycloaddition can proceed either by the overlapping of the highest occupied molecular orbital (HOMO) of a 1,3-dipole with the lowest unoccupied molecular orbital (LUMO) of a dipolarophile (left panel of Figure 1A), or, vice versa, by the overlapping of the LUMO of a 1,3-dipole with the HOMO of a dipolarophile (right panel of Figure 1A) [34]. A preferred path of HOMO-LUMO overlapping is determined by the difference in energies of the corresponding orbitals; the smaller difference favors the reaction [35]. Complementing the FMO theory with the distortion–interaction model allows one to explain the reactivity trends found in computational studies of the reactions of diazonium, nitrilium, and azomethine betaines with ethylene and acetylene [36]. According to this model, the activation energy *E_act_* for a bimolecular process is composed of the contributions of the distortion energies of the dipole and dipolarophile (*E_dist_*) and the energy of the interaction (*E_int_*) between distorted reactants (Figure 1B). It has been shown that the calculated *E_act_* values correlate with the distortion energies of reactants, which was explained by the major contribution of *E_dist_* to the overall energetics of the reaction [37,38].

In all, previous studies of 1,3-dipolar cycloadditions demonstrate a complex dependence of the reactivity on a structure of both addends. At the same time, most of these studies involve computationally obtained data for the gas-phase reactions. Thus, care must be exercised when applying the respective results to the bulk AAP process because they ignore the effect of continuously changing media on the thermodynamic properties of the reagents, products, and transition states, as typically found for bulk polymerization [39]. In this situation, experimental studies are the only source of reliable kinetic and mechanistic information.

**Figure 2:**
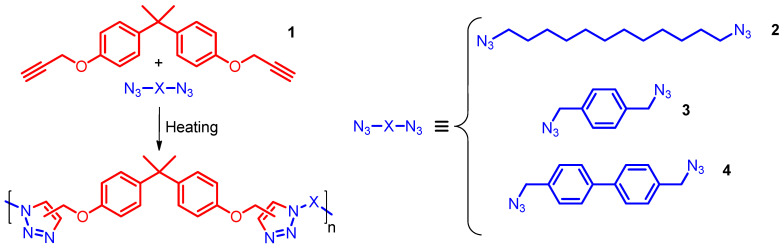
Scheme of A_2_/B_2_-type AAP and structures of studied monomers **1**–**4**.

The present study reports the results of the presumably first experimental quantitative investigation of the reactivity of organic azides in metal-free A_2_/B_2_-type AAP. It focuses on the bulk reaction of the dipropargyl ether of bisphenol A with three organic diazides **2**–**4** (Figure 2). The structures of the latter are chosen to probe the effect of the nature of the hydrocarbon spacer between functional groups (aliphatic vs. aromatic) on their reactivity in the polyaddition process. The kinetics of the polymerization is studied by differential scanning calorimetry (DSC) and parameterized with the aid of isoconversional and model-fitting approaches [40,41]. Obtained kinetic data are further used for quantitative assessment of the reactivity of the studied organic diazides. The evaluated difference in the reactivity of studied diazides is interpreted by the influence of the electronic and steric effects of hydrocarbon spacers. An effect of the azide structure on the properties of the respective polymers is studied and discussed.

## 2. Materials and Methods

### 2.1. Materials

The listed chemicals were applied directly as purchased or after additional purification. Acetonitrile (HPLC grade, ITW Reagents, Monza, Italy), 1,4-benzenedimethanol (>98%, Macklin, Shanghai, China), 4,4′-bis(chloromethyl)biphenyl (>96%, Aladdin, Shanghai, China), bisphenol A (>97%, Merck, Saint Louis, MO, USA), propargyl bromide (>97%, TCI, Tokyo, Japan), 1,12-dibromododecane (>96%, Acros Organics, France), dichloromethane (>99%, Chimmed, Moscow, Russia), *N*,*N*-dimethylformamide (DMF, >99%, EKOS-1, Moscow, Russia), diphenyl phosphoryl azide (>97%, Macklin, Shanghai, China), ethyl acetate (>99%, EKOS-1, Moscow, Russia), hexane (>99%, Khimprom-M, Yaroslavl, Russia), phosphorus(V) oxide (>99%, Vekton, Saint-Petersburg, Russia), potassium carbonate (>98%, TatChemProduct, Kazan, Russia), potassium hydroxide (>99%, Reakhim, France), silica gel (60 Å, 0.04–0.063 mm, Merck, Rahway, NJ, USA), sodium azide (>99%, Corvine Chemicals and Pharmaceuticals, Hyderabad, India), sodium sulfate (anhydrous, >99%, Khimprom-M, Yaroslavl, Russia), triethylamine (>99%, Fisher Chemical, Loughborough, UK) were used as received. Acetone (>99.5%, Khimprom-M, Yaroslavl, Russia) and tetrahydrofuran (THF, >99%, Chimmed, Moscow, Russia) were distilled over phosphorus(V) oxide and potassium hydroxide, respectively.

Synthesis of bisphenol A dipropargyl ether (**1**). An amount of 2.00 g (8.8 mmol) of bisphenol A was stirred with 3.13 g (26.3 mmol) of propargyl bromide and 6.05 g (43.8 mmol) of potassium carbonate in 25 mL of refluxing acetone for 48 h. After finishing, the solvent was evaporated, the residue was mixed with 10 mL of dichloromethane, and then the crude product was purified by column chromatography (silica gel, hexane–dichloromethane (10:1 by volume) as an eluent). After drying in a vacuum, the desired diether was obtained as a colorless viscous liquid with a yield of 95% (2.53 g). ^1^H NMR (400 MHz, CDCl_3_, δ, ppm): 7.17 (d, *J* = 8.7 Hz, 4H, CH aryl), 6.89 (d, *J* = 8.7 Hz, 4H, CH aryl), 4.67 (d, *J* = 2.2 Hz, 4H, OCH_2_C≡), 2.52 (t, *J* = 2.2 Hz, 2H, ≡CH), 1.65 (s, 6H, CH_3_). ^13^C NMR (101 MHz, CDCl_3_, δ, ppm): 155.6 (C aryl–O), 144.0 (C aryl), 127.9 (CH aryl), 114.3 (CH aryl), 78.9 (C≡CH), 75.5 (≡CH), 55.9 (OCH_2_C≡), 41.9 (C(CH_3_)_2_), 31.1 (CH_3_). IR (cm^−1^): 3288 (≡C–H, ν), 3039, 2967, 2932, 2871 (C–H, ν), 2121 (C≡C, ν), 1607, 1583, 1508 (C=C aryl, ν), 1454, 1365, 1298, 1264 (C–H, δ), 1222, 1183 (C–O–C, ν_as_), 1108 (C–H, δ), 1028 (C–O–C, ν_s_), 925, 830, 810 (C–H, δ), 683 (≡C–H, δ).

Synthesis of 1,12-diazidododecane (**2**). An amount of 3.00 g (9.1 mmol) of 1,12-dibromododecane was dissolved in 30 mL of DMF on heating. An amount of 1.78 g (27.4 mmol) of sodium azide was added to the hot solution, the synthesis proceeded with stirring at 95 °C for 24 h. After cooling, 30 mL of distilled water was poured in, and then the diazide was extracted by ethyl acetate (3 × 20 mL). The extract was washed with water (5 × 20 mL) and then dried with anhydrous sodium sulfate for 24 h. After solvent evaporation, the diazide was purified by column chromatography (silica gel, dichloromethane as an eluent). The product was dried in a vacuum, so the desired diazide **2** was produced as a colorless liquid with a yield of 98% (2.27 g). ^1^H NMR (400 MHz, CDCl_3_, δ, ppm): 3.25 (t, *J* = 7.0 Hz, 4H, NCH_2_), 1.64–1.54 (m, 4H, NCH_2_CH_2_), 1.42–1.21 (m, 16H, (CH_2_)_8_). ^13^C NMR (101 MHz, CDCl_3_, δ, ppm): 51.6 (NCH_2_), 29.6, 29.6, 29.3, 29.0, 26.8 ((CH_2_)_10_). IR (cm^−1^): 2928, 2856 (C–H, ν), 2094 (–N=N^+^=N^−^, ν), 1465, 1349 (C–H, δ), 1259 (C–N, ν), 723 (C–H, δ).

Synthesis of 1,4-bis(azidomethyl)benzene (**3**)**.** An amount of 7.17 g (26.1 mmol) of diphenyl phosphoryl azide was added to a solution of 1.50 g (10.9 mmol) of 1,4-benzenedimethanol in 30 mL of THF. The mixture was cooled down to 0 °C under argon flow, and 2.64 g (26.1 mmol) of triethylamine was poured in. The reaction proceeded at room temperature under argon atmosphere and with stirring for 48 h. As the synthesis completed, the solvent was evaporated, and then the target product was separated by column chromatography (silica gel, dichloromethane as an eluent). The diazide was dried in a vacuum, resulting in a yellowish liquid with a yield of 73% (1.50 g). ^1^H NMR (400 MHz, CDCl_3_, δ, ppm): 7.35 (s, 4H, CH aryl), 4.36 (s, 4H, NCH_2_). ^13^C NMR (101 MHz, CDCl_3_, δ, ppm): 135.6 (C aryl–CH_2_), 128.7 (CH aryl), 54.5 (NCH_2_). IR (cm^−1^): 3032, 2940, 2881 (C–H, ν), 2096 (–N=N^+^=N^−^, ν), 1516 (C=C aryl, ν), 1445, 1421, 1345 (C–H, δ), 1254 (C–N, ν), 1207, 1114, 1022, 880, 805, 755, 669 (C–H, δ).

Synthesis of 4,4′-bis(azidomethyl)biphenyl (**4**)**.** An amount of 1.90 g (7.6 mmol) of 4,4′-bis(chloromethyl)biphenyl was dissolved in 20 mL of DMF, the mixture was stirred with 1.97 g (30.3 mmol) of sodium azide at 95 °C for 24 h. Further, the target diazide was extracted from the cooled mixture by adding 30 mL of water and 20 mL of ethyl acetate (repeated trice), each time separating the organic phase. The crude extract was washed by excess of water (5 × 20 mL), dried with anhydrous sodium sulfate for 24 h. The product was concentrated by solvent evaporation, purified by column chromatography (silica gel, dichloromethane as an eluent), and then dried in a vacuum. The diazide **4** formed white crystals with a yield of 96% (1.92 g). ^1^H NMR (400 MHz, CDCl_3_, δ, ppm): 7.62 (d, *J* = 8.1 Hz, 4H, CH aryl), 7.40 (d, *J* = 8.1 Hz, 4H, CH aryl), 4.40 (s, 4H, NCH_2_). ^13^C NMR (101 MHz, CDCl_3_, δ, ppm): 140.7 (C aryl–C aryl), 134.8 (C aryl–CH_2_), 128.9, 127.7 (CH aryl), 54.7 (NCH_2_). IR (cm^−1^): 3034, 2912, 2850 (C–H, ν), 2217, 2114 (–N=N^+^=N^−^, ν), 1563, 1501 (C=C aryl, ν), 1437, 1401, 1358, 1319 (C–H, δ), 1288 (C–N, ν), 1143, 1002, 940, 828, 802, 658 (C–H, δ). mp. 70.5 °C (lit. value 70–71 °C [42]).

### 2.2. Methods

HPLC analyses were performed on a Dionex UltiMate 3000 (Thermo Fischer Scientific, Waltham, MA, USA) chromatograph furnished with a UV detector (210 nm) and Dionex Acclaim 120 chromatographic column (C_18_-bonded silica, 5 μm, 120 Å, 4.6 × 150 mm). A mixture of acetonitrile (85 vol. %) and deionized water (15 vol. %) was applied as an eluent at a flow rate of 0.9 mL min^−1^. Gel permeation chromatography (GPC) measurements were executed on a Dionex UltiMate 3000 (Thermo Fischer Scientific, Waltham, MA, USA) chromatograph supplemented by a refractive index detector RefractoMax 520 and PLgel Agilent Mixed-D column. THF was used as an eluent at a flow rate of 1 mL min^−1^. Polystyrene standards were utilized for the GPC calibration. ^1^H and ^13^C NMR analyses were implemented on a Bruker AVANCE III NMR spectrometer functioning at 400 MHz (^1^H) and 101 MHz (^13^C) with CDCl_3_ or DMSO-*d*_6_ as solvents. Chemical shifts are stated in delta (δ) units in parts per million (ppm). ^1^H NMR spectra of polytriazoles were obtained for the soluble samples with incomplete conversion (>97%). IR spectral data were recorded on a Bruker Vertex 70 FTIR spectrometer. DSC experiments were performed on a DSC 3+ (Mettler-Toledo) heat flux instrument in the atmosphere of argon flow (80 mL min^−1^). Temperature, heat flow, and tau-lag calibrations were implemented with the aid of indium and zinc standards. Kinetic measurements were carried out in the temperature range from 25 to 250 °C at the heating rates of 0.5, 1.0, 2.0, and 4.0 °C min^−1^. Samples for the DSC experiments were prepared by mixing equimolar quantities of respective diazide **2**–**4** and bisphenol A dipropargyl ether **1** in a glass vial. The samples were weighed into 40 μL aluminum pans and hermetically sealed in argon atmosphere. The mass of the sample for each run was 5.0 ± 0.5 mg. Glass transition temperatures were measured in the temperature range from −30 to 250 °C at a heating rate of 10.0 °C min^−1^ in 40 μL aluminum pans. Thermogravimetric measurements were conducted on STA 449 F1 Jupiter (Netzsch) apparatus under argon flow (75 mL min^−1^) in the temperature range from 40 to 1000 °C at a heating rate of 10 °C min^−1^. The samples were weighed into 100 µL corundum crucibles with pierced lids. The average mass of the sample for each run was 5.0 ± 0.5 mg.

## 3. Computations

The reaction rate of a thermally stimulated process, $r\left(\alpha ,T\right),$ is parameterized in terms of the temperature, *T*, and the extent of conversion, *α,* as follows:

(1) $$r(\alpha ,T)=\frac{d\alpha }{dt}=k\left(T\right)f\left(\alpha \right),$$

where *t* is the time, *f(α)* is the reaction model, and *k(T)* is the rate constant. The latter is represented by the Arrhenius equation:

(2) $$k\left(T\right)=A\;exp\left[\frac{-E}{RT}\right],$$

where *E* is the activation energy, *A* is the preexponential factor, and *R* is the gas constant.

The activation energies and preexponential factors were calculated isoconversionally in accordance with the ICTAC Kinetic Committee recommendations [41,43,44]. The values of *α* were determined from the DSC peaks as the partial areas. The isoconversional activation energy, *E_α_*, for the liquid-state polymerization studied in non-isothermal conditions was determined by the flexible integral isoconversional method of Vyazovkin [45,46,47]. Unlike simpler (rigid) integral methods, it eliminates the systematic error in *E_α_* that arises when *E_α_* varies significantly with *α* [45]. The error is eliminated by performing the piecewise integration that assumes the constancy of *E_α_* over a very narrow integration range, Δ*α*. In this study the value of Δ*α* was kept as 0.01. For each Δ*α*, *E_α_* was found by minimizing the function:

(3) $$\Psi \left(E_{\alpha }\right)=\sum_{i=1}^{p}\sum_{j\neq i}^{p}\frac{J\left[E_{\alpha },T_{i}\left(t_{\alpha }\right)\right]}{J\left[E_{\alpha },T_{j}\left(t_{\alpha }\right)\right]}$$

where

(4) $$J\left[E_{\alpha },T_{i}\left(t_{\alpha }\right)\right]=\int_{t_{\alpha -∆\alpha }}^{t_{\alpha }}\exp\left[\frac{-E_{\alpha }}{RT_{i}\left(t\right)}\right]dt$$

And *p* is the number of the temperature programs, *T*(*t*). The uncertainties in the *E_α_* values were estimated as described in ref. [48].

The reactivity of compounds participating in *n*th-order reactions (e.g., reactions *A* and *B*) studied under non-isothermal conditions can be quantified by the reactivity factor *Z_T_*(*A*/*B*) expressed by Equation (5) [49]. The notation *A*/*B* means that the reactivity of compounds in reaction *A* is evaluated relative to those in *B*. The reactivity of compounds in the reference process is taken conditionally as unity for any conversion and temperature value.

(5) $$Z_{\;T}(A/B)=Z_{\;\alpha ,T}\frac{C_{0,B}^{n_{B}-1}}{C_{0,A}^{n_{A}-1}}{\left(1-\alpha \right)}^{n_{B}-n_{A}}$$

where

(6) $$Z_{\alpha ,\;T}(A/B)=\frac{J\left[E_{\alpha ,A},T_{i}\left(t_{\alpha }\right)\right]}{J\left[E_{\alpha ,B},T_{j}\left(t_{\alpha }\right)\right]}exp\left[-\frac{{(E}_{\alpha ,B}-E_{\alpha ,A})}{RT}\right]$$

The activation entropy ($\Delta S^{\neq }$) for the process in the solution was calculated from the preexponential factor *A* value according to the activated complex theory [50]:

(7) $$\Delta S^{\neq }=R\left[\ln\left(\frac{A\;h}{k_{B}T}\right)-1\right]$$

where *h* and *k_B_*, respectively, are the Planck and Boltzmann constants.

## 4. Results and Discussion

**Figure 3:**
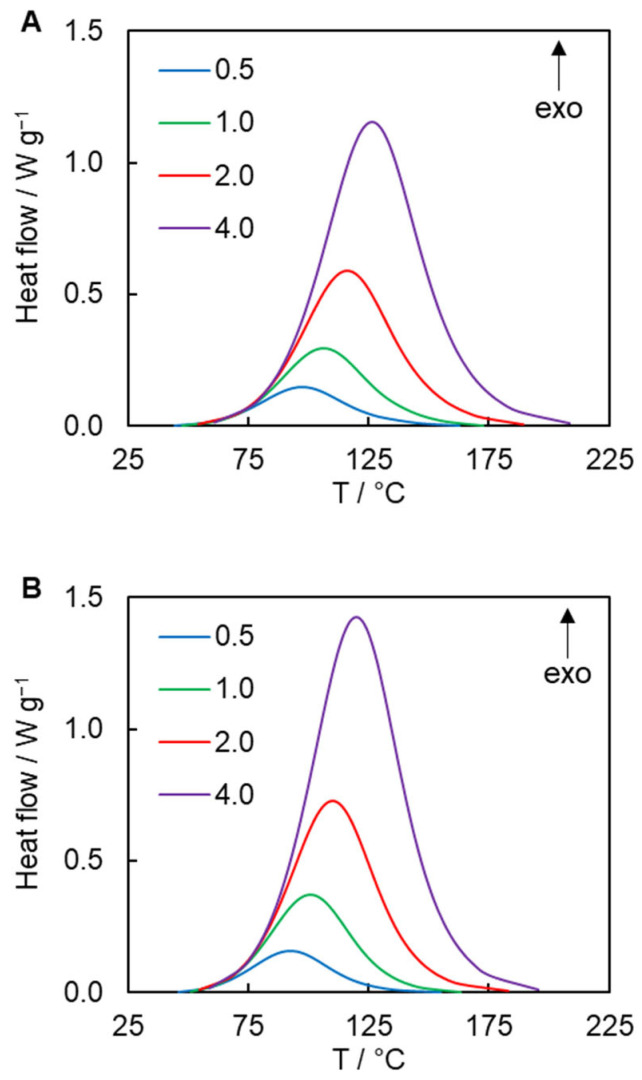

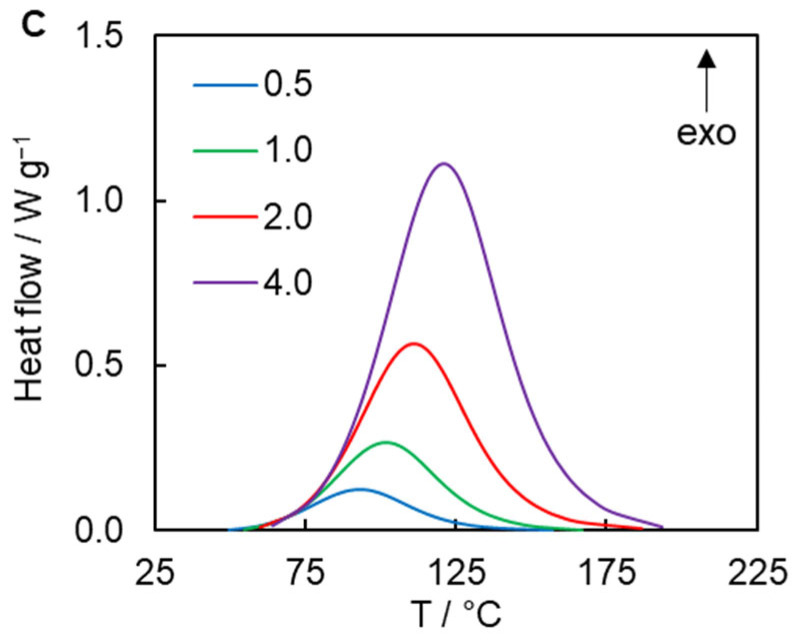
Heat flow curves for the polymerization of dipropargyl ether of BPA **1** (**A**), **2** (**B**), and **3** (**C**) performed at the heating rates of 0.5 (blue), 1.0 (green), 2.0 (red), 4.0 (purple) °C min^−1^.

AAP is a highly exothermic process generating 200–270 kJ mol^−1^ of heat due to the formation of stable aromatic triazoles (Figure 2) [23,51,52,53]. Thus, the kinetics of this reaction can be conveniently studied by calorimetric techniques, e.g., by differential scanning calorimetry (DSC). To avoid possible vitrification of the reaction mixtures, polymerization of all systems has been studied in non-isothermal conditions. Figure 3 presents the DSC curves for the reactions under study measured at 0.5, 1.0, 2.0, and 4.0 °C min^−1^. Relatively slow heating rates have to be used to avoid thermal decomposition of organic azides [52]. A comparison of the heat flow curves shows that the peak temperatures for the polymerization of diazides **3** and **4** are shifted to lower values by ~4–7 °C relative to the reaction with aliphatic diazide **2.** This means that the former two reactions need less thermal stimulation (heat) than the latter one. Since all studied reaction systems contain the same dialkyne **1**, it is logical to assume that the lower need for thermal stimulation is associated with the higher reactivity of diazides **3** and **4** compared to that of **2**. A quantitative assessment of the reactivity of the studied organic azides is discussed further. The measured average heats of the reaction are 238 ± 11, 231 ± 25, and 215 ± 23 kJ mol^−1^ (values are normalized to 1 mole of equimolar mixture) for the polymerization of dialkyne **1** with organic diazides **2–4**, respectively. The obtained values agree with the aforementioned range of the reaction heats.

In general, AAP is not regioselective and proceeds with the concurrent formation of isomeric triazole units randomly alternating in the polymer chain [53]. Therefore, from a kinetic point of view, the studied systems represent a process consisting of two concurrent irreversible reactions. In the case of two concurrent reactions with distinctly different activation energies, *E*_1_ and *E*_2_, the effective activation energy of the overall process should monotonically vary between these two values [54]. However, recently, we have found that the formation of isomeric 1,2,3-triazoles in azide–alkyne cycloaddition as well as in AB-type polyaddition is characterized by similar activation energies, i.e., *E*_1_ ≈ *E*_2_ [52,53]. Also, considering that the elementary steps of both reactions differ only by the orientation of the reactants, it is natural to assume that the reaction order *n* of both reactions is also the same. In this case the overall reaction kinetics can be simplified to a single *n*th-order reaction rate Equation (8) [52]:

(8) $$\frac{d\alpha }{dt}=A_{ef}\exp\left[-\frac{E}{RT}\right]{\left(1-\alpha \right)}^{n}$$

where

(9) $${{A_{ef}=C}_{0}^{n-1}(A}_{1}+\;A_{2})$$

*C*_0_ is the initial concentration of the azide and alkyne functional groups in an equimolar mixture of the monomers, *A_ef_* is the effective preexponential factor characterizing the overall process, *A*_1_ and *A*_2_ are the preexponential factors characterizing individual concurrent reactions, *E* is the activation energy of the overall process, and *n* is a reaction order. In Equation (9) the *C*_0_ value is dimensionless since it is taken relative to the standard state (i.e., 1 mol L^−1^). This helps to avoid confusion with the dimensions of the preexponential factors for the processes with fractional reaction orders, which are often encountered in experimental kinetics [55].

**Figure 4:**
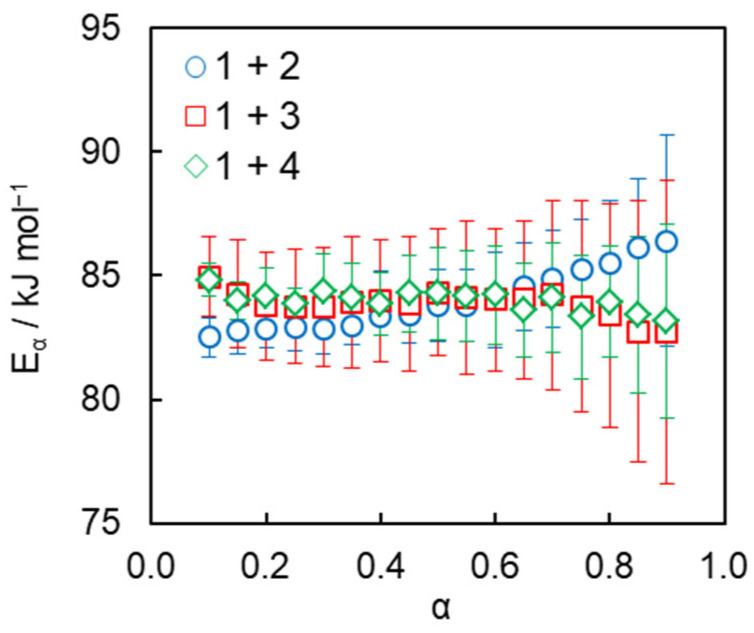
Dependencies of the effective activation energy on conversion for studied polyaddition processes.

The aforementioned closeness of the activation energies of concurrent reactions is also found in the present study for A_2_/B_2_-type AAP. It is revealed by the isoconversional analysis of the obtained calorimetric data that demonstrates the absence of any noticeable variation in *E_α_* in a whole conversion range (Figure 4) for all studied systems. This makes Equation (8) fully applicable for the parametrization of the studied reactions. The average values of the activation energy are determined to be 84 kJ mol^−1^ for all three studied systems. Similar values of the activation energy were reported previously for bulk azide–alkyne cycloaddition and polyaddition reactions [19,25,52].

It is worth noting that for the processes described by Equations (8) and (9), the ratio of concurrent reaction products is determined by the ratio of respective preexponential factors, i.e., by Equation (10):

(10) $$\gamma =\frac{A_{1}}{A_{2}}$$

As seen from Equation (10), the ratio *γ* is independent of the temperature because the activation energies of both concurrent reactions are practically equal. Thus, evaluating the ratio of the isomeric products permits the preexponential factors *A*_1_ and *A*_2_ of the individual concurrent reactions to be estimated from the effective overall value obtained from the calorimetric measurements.

**Figure 5:**
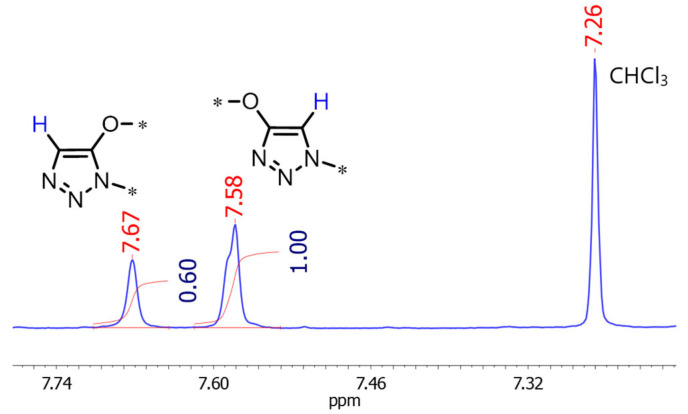
Fragment of ^1^H NMR spectrum of polymerization product of dialkyne **1** and diazide **2**. Asterisk sign denotes the positions where the polymer chain continues.

^1^H NMR spectroscopy is conveniently used for evaluating the ratio of isomeric triazolic fragments in polymer chains. For the quantitative assessment of *γ,* we have used the integral intensities of the resonance signals for the C4-H and C5-H protons of the isomeric 1,2,3-triazolic units (Figure 5, Figures S1–S3 in Supplementary Information file). The resulting *γ* values are presented in Table 1. The obtained ratios of the isomers are similar to the previously reported ones for metal-free azide–alkyne cycloaddition and polyaddition [52,56].

The kinetic parameters of the AAP are readily determined by fitting Equation (8) directly to the transformation rate data. To avoid the compensation effect between the effective preexponential factor and activation energy, the latter one is fixed at the value equal to the respective mean value of *E_α_* (Figure 4). The *A_ef_* and *n* values have been optimized during fitting. The previously evaluated *γ* and *C*_0_ values have been further employed for calculating the individual preexponential factor values *A*_1_ and *A*_2_ by Equations (9) and (10). The resulting kinetic parameters of the reaction are presented in Table 1. Since azide–alkyne cycloaddition is a bimolecular reaction, it is natural to expect that the reaction order *n* has to be equal to 2. However, polyaddition is inevitably accompanied by monomolecular macrocyclizations of intermediate oligomers [17]. These reactions also contribute to the measured heat flow; therefore, the values of *n* experimentally determined from DSC may be lower than 2.

For quantitative assessment of the reactivity, we apply two approaches. The first one is based on a recently developed isoconversional approach that permits calculating a dependence of the reactivity on the conversion [49]. Equations (5) and (6) express this approach applied to *n*th-order reactions. The second approach is based directly on the IUPAC’s definition of the reactivity as a relative kinetic property measured at some specific conditions [57]. In this case the ratio of two effective rate constants, which have the form of Equation (2) with *A = A*_1_ *+ A*_2_, can be used as a numerical expression of the reactivity [58] (Equation (11)). Because the activation energies for all our reactions are practically the same, the exponential terms of the respective rate constants are also the same for any given temperature. Then, for all studied processes, the reactivity *Z_T_* can be simply calculated as the ratio of the sum of the preexponential factors for parallel reactions in processes A and B (Equation (11)):

(11) $$Z_{\;T}(A/B)=\frac{{k\left(T\right)}_{A}}{{k\left(T\right)}_{B}}=\frac{{{(A}_{1}+\;A_{2})}_{A}}{{{(A}_{1}+\;A_{2})}_{B}}$$

In our further calculations we will use the least reactive diazide **2** as a reference. This means that its reactivity equals unity by definition, whereas the reactivities of the two other diazides **3** and **4** are evaluated relative to it.

**Figure 6:**
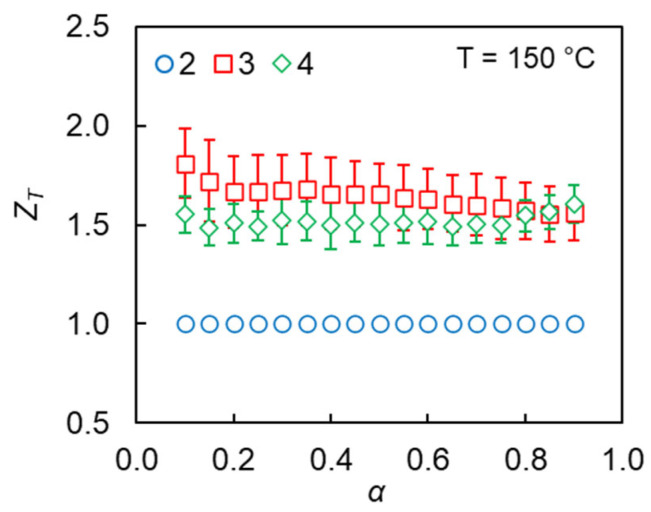
Dependencies of the reactivity of diazides **2**–**4** on conversion in AAP.

The dependencies of *Z_T_* on the conversion calculated by the isoconversional approach are presented in Figure 6. As one can see, diazides **3** and **4** are ~1.6 times more reactive than diazide **2**. Table 2 contains the averaged *Z_T_* values calculated by the isoconversional approach and the ones calculated by Equation (11). Clearly, both approaches yield the same results within the uncertainties of the calculations.

**Figure 7:**
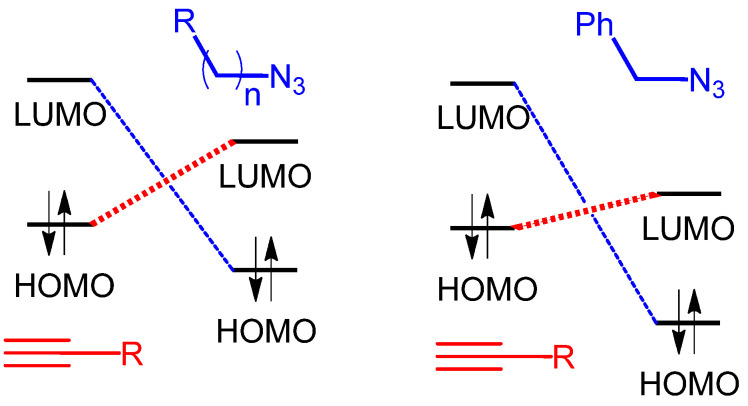
Schematic interactions between molecular orbitals of alkyne with alkyl azide and benzyl azide. Bold dash lines denote preferable interactions.

The increased reactivity of diazides **3** and **4** compared to that of **2** can be explained by the difference in the electronic effects of the alkyl and benzyl fragments attached to the azide groups. According to the FMO theory, the higher electron-donating ability of the alkyl group compared to the benzyl one should increase the energy of the HOMO and LUMO of the azide group. Assuming that the reaction proceeds by the overlapping of the HOMO (alkyne) and LUMO (azide), the higher energy of the azide **2** LUMO results in a larger energetic gap between the frontier molecular orbitals (Figure 7), which disfavors the reaction kinetics and makes azide **2** less reactive than **3** and **4**. It should be noted that in the FMO theory, a higher (lower) reactivity means a higher (lower) rate constant, i.e., the theory does not link the reactivity to more specific parameters, like the activation energy, preexponential factor, etc. [59].

On the other hand, the activated complex theory [46] can explain the difference in the reactivity in a more quantitative manner. Since the activation energy for the reactions of **2**, **3**, and **4** is practically identical, the lower reactivity of **2** compared to **3** and **4** is associated with the smaller preexponential factor (Table 1). Mechanistically, this can be interpreted by the difference in the steric availability of azide groups. For example, one can assume that in the case of diazides **3** and **4**, the reacting groups can acquire a preferential alignment with alkyne promoted by the π-π stacking between the aromatic fragments of the reacting monomers. No such alignment is possible in the case of the aliphatic monomer **2.** This difference should reveal itself in a larger activation entropy for **3** and **4**. Since the activation entropy of bimolecular reactions is normally negative, its larger value corresponds to a less negative number. Indeed, the activation entropy values calculated by Equation (7) for the reactions involving diazides **3** and **4** turn out to be ~4 J K^−1^ mol^−1^ less negative than those for **2**.

The properties of the polymerization products are reported in Table 3. The difference in the molecular mass distributions of the obtained polymers evaluated by GPC is striking, but can be related to the reduced solubility of the polymerization products based on azides **3** and **4**. Quite likely only the low-molecular-weight fraction of the polymers based on diazides **3** and **4** dissolves in THF, which results in underestimated values of the molecular mass. The thermal stability of the polyaddition products in an inert atmosphere has been evaluated by thermogravimetry as the temperature to reach 5% decomposition, T_d5%_. Respectively, the thermal stability appears slightly higher for the **1** + **2** polymerization product. In turn, the presence of the aromatic fragments, which are more resistant to thermal decomposition compared to the alkyl ones, significantly increases the char yield. The higher content of the rigid aromatic fragments can also enhance interchain π-π interactions, which explains the higher glass transition temperatures, *T_g,_* of poly(1,2,3-triazole)s based on diazides **3** and **4**. Note that an earlier study on the copper-catalyzed polymerization of the same monomers reports similar values of *T_g_* for polymers based on diazides **2** and **3** [60].

**Figure 8:**
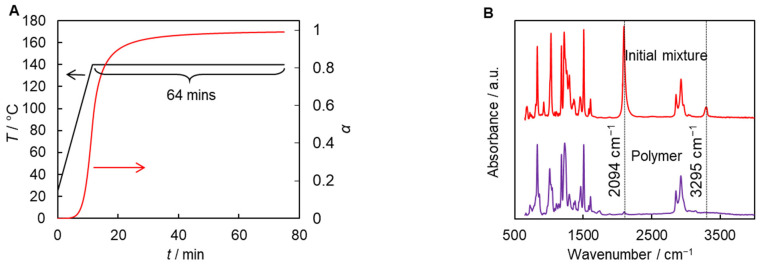
Predicted conversion curve (red line) for AAP between dialkyne **1** and diazide **2** calculated for chosen temperature program (black line) (**A**). FTIR spectra of initial **1** + **2** mixture and corresponding polymerization product (**B**).

The obtained kinetic parameters of AAP and the measured thermal properties of the resulting polymers have been employed further for predicting the isothermal conditions suitable for the synthesis of all three polymers. We can base such estimates on the kinetic parameters of AAP for the least reactive system, i.e., dialkyne **1** + diazide **2** (Table 1), assuming that if at some temperatures this system reacts to completion, the other two would do too. At the same time, isothermal polymerization has to be carried out at a temperature higher than the limiting glass transition temperature of the resulting polymers (i.e., *T_iso_* > 136 °C). This allows one to avoid vitrification of the reaction mixture and ensure complete conversion of monomers [61]. The calculations based on the *n*th-order reaction model (Equation (8)) predict that 99% of the monomer conversion will be achieved during isothermal polymerization at 140 °C for 64 min preceded by linear heating to this target temperature at 10 °C min^−1^ (Figure 8A). FTIR spectroscopy of the polymer samples synthesized according to this temperature program shows complete disappearance of the absorption bands at 3295 cm^−1^ corresponding to the terminal C-H group of the dialkyne **1** and of azide groups at 2094 cm^−1^, which confirms the completeness of the polymerization process (Figure 8B).

## 5. Conclusions

The kinetics of metal-free A_2_/B_2_-type azide–alkyne polyaddition has been studied in detail. Varying the organic diazides makes it possible to probe the effect of the nature of a hydrocarbon spacer between the functional groups (aliphatic vs. aromatic) on their reactivity. It is found that the formation of isomeric 1,2,3-triazolic fragments in the polyaddition process is characterized by the same activation energy of 84 kJ mol^−1^ for all diazides. The reactivity of diazides in polyaddition is quantified in the entire conversion range by a recently developed isoconversional approach. Monomers containing benzyl azide groups demonstrate ~1.6 times higher reactivity than that of the monomers with alkyl azide groups. The difference in the reactivity is explained by the difference in the electronic effects of hydrocarbon spacers as well as by the difference in the steric availability of the reacting groups within the FMO and activated complex theories, respectively. The presence of aromatic fragments in the diazide structure results in distinctly higher values of *T_g_* and char yield, whereas the thermal stability becomes insignificantly lower. The obtained kinetic parameters are applicable to the prediction of the universal isothermal conditions suitable for the synthesis of all three polymers.

## Figures and Tables

**Table 1:** Kinetic parameters for polyaddition of monomers **1–4**.

Sample	*γ*	*^a^ C*_0_/mol L^−1^	*^b^ E*/kJ mol^−1^	*A*_1_/s^−1^	*A*_2_/s^−1^	*n*
**1** + **2**	1.63	3.6	84 ± 2	(0.97 ± 0.03) × 10^8^	(0.59 ± 0.02) × 10^8^	1.72 ± 0.02
**1** + **3**	1.56	4.1	84 ± 3	(1.54 ± 0.04) × 10^8^	(0.99 ± 0.03) × 10^8^	1.61 ± 0.02
**1** + **4**	1.56	3.5	84 ± 2	(1.50 ± 0.03) × 10^8^	(0.96 ± 0.02) × 10^8^	1.65 ± 0.02

*^a^* Initial concentrations of azide and alkyne groups in equimolar mixture of reagents. *^b^* Value kept constant during fitting.

**Table 2:** Reactivity of diazides **2**–**4** in polyaddition process at 150 °C.

Diazide	*Z_T_* (Equation (5))	*Z_T_* (Equation (11))
**2**	1.00	1.00
**3**	1.64 ± 0.13	1.62 ± 0.16
**4**	1.52 ± 0.08	1.58 ± 0.10

**Table 3:** Characteristics of polymerization products.

Reacting System	M_w_/kDa	M_n_/kDa	PDI	T_d5%_/°C	*^a^* Char/%	T_g_/°C
**1** + **2**	45.5	22.6	2.02	358	10.5	32
**1** + **3**	9.3 *^b^*	4.5 *^b^*	2.08 *^b^*	347	37.0	122
**1** + **4**	11.2 *^b^*	7.4 *^b^*	1.51 *^b^*	350	33.1	136

*^a^* Char yields are evaluated at 1000 °C. *^b^* Partial solubility of polymer.

## Data Availability

The data presented in this study are available on reasonable request from the corresponding authors.

## Supplementary Materials

The following supporting information can be downloaded at: https://www.mdpi.com/article/10.3390/polym17141909/s1, Figure S1: ^1^H NMR spectrum of polymerization product of dialkyne **1** and diazide **2**; Figure S2: ^1^H NMR spectrum of polymerization product of dialkyne **1** and diazide **3**; Figure S3: ^1^H NMR spectrum of polymerization product of dialkyne **1** and diazide **3**.

## Abbreviations

The following abbreviations are used in this manuscript:

AAP	Azide–alkyne polyaddition
AAC	Azide–alkyne cycloaddition
HOMO	Highest occupied molecular orbital
LUMO	Lowest unoccupied molecular orbital
DSC	Differential scanning calorimetry
FMO	Frontier molecular orbital

## References

[1] Wang K., Amin K., An Z., Cai Z., Chen H., Chen H., Dong Y., Feng X., Fu W., Gu J. (2020). Advanced functional polymer materials. Mater. Chem. Front..

[2] Jin Y., Joshi M., Araki T., Kamimura N., Masai E., Nakamura M., Michinobu T. (2023). Click Synthesis of Triazole Polymers Based on Lignin-Derived Metabolic Intermediate and Their Strong Adhesive Properties to Cu Plate. Polymers.

[3] E Y., Wan L., Huang F., Du L. (2013). New heat-resistant polytriazole adhesives: Investigation of adhesion of polytriazole resins to metals. J. Adhes. Sci. Technol..

[4] Fang J., Wan L., Wang L., Bie R., Zhang Y., Huang F. (2021). Preparation and performance of urethane-modified polytriazole adhesives. Polym. Eng. Sci..

[5] Armelin E., Whelan R., Martínez-Triana Y.M., Alemán C., Finn M.G., Díaz D.D. (2017). Protective Coatings for Aluminum Alloy Based on Hyperbranched 1,4-Polytriazoles. ACS Appl. Mater. Interfaces.

[6] Kantheti S., Sarath P.S., Narayan R., Raju K.V.S.N. (2013). Synthesis and characterization of triazole rich polyether polyols using click chemistry for highly branched polyurethanes. React. Funct. Polym..

[7] Sykam K., Donempudi S., Basak P. (2022). 1,2,3-Triazole rich polymers for flame retardant application: A review. J. Appl. Polym. Sci..

[8] Döhler D., Michael P., Binder W.H. (2017). CuAAC-Based Click Chemistry in Self-Healing Polymers. Acc. Chem. Res..

[9] Wang X., Hu R., Zhao Z., Qin A., Tang B.Z. (2016). Self-healing hyperbranched polytriazoles prepared by metal-free click polymerization of propiolate and azide monomers. Sci. China Chem..

[10] Wei Q., Wang J., Shen X., Zhang X.A., Sun J.Z., Qin A., Tang B.Z. (2013). Self-healing hyperbranched poly(aroyltriazole)s. Sci. Rep..

[11] Ragin Ramdas M., Reghunadhan Nair C.P., Santhosh Kumar K.S. (2017). H-bonded polytriazoles: Synthesis and thermoresponsive shape memory properties. Eur. Polym. J..

[12] Obadia M.M., Jourdain A., Cassagnau P., Montarnal D., Drockenmuller E. (2017). Tuning the Viscosity Profile of Ionic Vitrimers Incorporating 1,2,3-Triazolium Cross-Links. Adv. Funct. Mater..

[13] Obadia M.M., Mudraboyina B.P., Serghei A., Montarnal D., Drockenmuller E. (2015). Reprocessing and Recycling of Highly Cross-Linked Ion-Conducting Networks through Transalkylation Exchanges of C–N Bonds. J. Am. Chem. Soc..

[14] Obadia M.M., Crépet A., Serghei A., Montarnal D., Drockenmuller E. (2015). Expanding the structural variety of poly(1,2,3-triazolium)s obtained by simultaneous 1,3-dipolar Huisgen polyaddition and N-alkylation. Polymer.

[15] Besset C., Binauld S., Ibert M., Fuertes P., Pascault J.-P., Fleury E., Bernard J., Drockenmuller E. (2010). Copper-Catalyzed vs Thermal Step Growth Polymerization of Starch-Derived α-Azide−ω-Alkyne Dianhydrohexitol Stereoisomers: To Click or Not To Click?. Macromolecules.

[16] Tsarevsky N.V., Sumerlin B.S., Matyjaszewski K. (2005). Step-Growth “Click” Coupling of Telechelic Polymers Prepared by Atom Transfer Radical Polymerization. Macromolecules.

[17] Binauld S., Damiron D., Hamaide T., Pascault J.-P., Fleury E., Drockenmuller E. (2008). Click chemistry step growth polymerization of novel α-azide-ω-alkyne monomers. Chem. Commun..

[18] Binauld S., Fleury E., Drockenmuller E. (2010). Solving the loss of orthogonality during the polyaddition of α-azide-ω-alkyne monomers catalyzed by Cu(PPh3)3Br: Application to the synthesis of high-molar mass polytriazoles. J. Polym. Sci. A Polym. Chem..

[19] Petrov A., Malkov G., Karpov S., Shastin A., Bakeshko A. (2019). Kinetic Study of the Polyaddition of Azide-Alkyne AB2 Monomers in Nonisotermic Conditions. Key Eng. Mater..

[20] Min B.S., Kim S.Y. (2017). Kinetics of in situ robust chain-ends crosslinked polymeric networks formed using catalyst- and solvent-free Huisgen cycloaddition reaction. Macromol. Res..

[21] Sheng X., Mauldin T.C., Kessler M.R. (2010). Kinetics of bulk azide/alkyne “click” polymerization. J. Polym. Sci. A Polym. Chem..

[22] Kargarfard N., Diedrich N., Rupp H., Döhler D., Binder W.H. (2018). Improving Kinetics of “Click-Crosslinking” for Self-Healing Nanocomposites by Graphene-Supported Cu-Nanoparticles. Polymers.

[23] Gorman I.E., Willer R.L., Kemp L.K., Storey R.F. (2012). Development of a triazole-cure resin system for composites: Evaluation of alkyne curatives. Polymer.

[24] Schlimbach J., Ogale A., Advani S.G., Hsiao K.-T. (2012). 14—Out-of-autoclave curing process in polymer matrix composites. Manufacturing Techniques for Polymer Matrix Composites (PMCs).

[25] Sheng X., Rock D.M., Mauldin T.C., Kessler M.R. (2011). Evaluation of different catalyst systems for bulk polymerization through “click” chemistry. Polymer.

[26] Huang D., Liu Y., Qin A., Tang B.Z. (2019). Structure–Property Relationship of Regioregular Polytriazoles Produced by Ligand-Controlled Regiodivergent Ru(II)-Catalyzed Azide–Alkyne Click Polymerization. Macromolecules.

[27] Qin A., Lam J.W.Y., Jim C.K.W., Zhang L., Yan J., Häussler M., Liu J., Dong Y., Liang D., Chen E. (2008). Hyperbranched Polytriazoles: Click Polymerization, Regioisomeric Structure, Light Emission, and Fluorescent Patterning. Macromolecules.

[28] Huang D., Liu Y., Qin A., Tang B.Z. (2023). Nickel-Catalyzed Azide–Alkyne Click Polymerization toward 1,5-Regioregular Polytriazoles. Macromolecules.

[29] Osawa Z. (1988). Role of metals and metal-deactivators in polymer degradation. Polym. Degrad. Stab..

[30] Deng F., Xu B., Gao Y., Liu Z., Yang D., Li H. (2012). Metal- and solvent-free, clickable synthesis and postpolymerization functionalization of poly(triazole)s. J. Polym. Sci. A Polym. Chem..

[31] Sustmann R., Trill H. (1972). Substituent Effects in 1,3-Dipolar Cycloadditions of Phenyl Azide. Angew. Chem. Int. Ed..

[32] Huisgen R. (1976). 1,3-Dipolar cycloadditions. 76. Concerted nature of 1,3-dipolar cycloadditions and the question of diradical intermediates. J. Org. Chem..

[33] Huisgen R. (1968). Mechanism of 1,3-dipolar cycloadditions. Reply. J. Org. Chem..

[34] Sustmann R. (1974). Orbital energy control of cycloaddition reactivity. Pure Appl. Chem..

[35] Geittner J., Huisgen R., Sustmann R. (1977). Kinetics of 1,3-dipolar cycloaddition reactions of diazomethane; A correlation with homo-lumo energies. Tetrahedron Lett..

[36] Breugst M., Reissig H.-U. (2020). The Huisgen Reaction: Milestones of the 1,3-Dipolar Cycloaddition. Angew. Chem. Int. Ed..

[37] Ess D.H., Houk K.N. (2007). Distortion/Interaction Energy Control of 1,3-Dipolar Cycloaddition Reactivity. J. Am. Chem. Soc..

[38] Ess D.H., Houk K.N. (2008). Theory of 1,3-Dipolar Cycloadditions: Distortion/Interaction and Frontier Molecular Orbital Models. J. Am. Chem. Soc..

[39] Vyazovkin S. (2015). Isoconversional Kinetics of Thermally Stimulated Processes.

[40] Vyazovkin S., Achilias D., Fernandez-Francos X., Galukhin A., Sbirrazzuoli N. (2022). ICTAC Kinetics Committee recommendations for analysis of thermal polymerization kinetics. Thermochim. Acta.

[41] Vyazovkin S., Burnham A.K., Favergeon L., Koga N., Moukhina E., Pérez-Maqueda L.A., Sbirrazzuoli N. (2020). ICTAC Kinetics Committee recommendations for analysis of multi-step kinetics. Thermochim. Acta.

[42] Tang J., Wan L., Zhou Y., Ye L., Zhou X., Huang F. (2017). Synthesis and performance study of a novel sulfonated polytriazole proton exchange membrane. J. Solid State Electrochem..

[43] Vyazovkin S., Chrissafis K., Di Lorenzo M.L., Koga N., Pijolat M., Roduit B., Sbirrazzuoli N., Suñol J.J. (2014). ICTAC Kinetics Committee recommendations for collecting experimental thermal analysis data for kinetic computations. Thermochim. Acta..

[44] Vyazovkin S., Burnham A.K., Criado J.M., Perez-Maqueda L.A., Popescu C., Sbirrazzuoli N. (2011). ICTAC Kinetics Committee recommendations for performing kinetic computations on thermal analysis data. Thermochim. Acta.

[45] Vyazovkin S. (2001). Modification of the integral isoconversional method to account for variation in the activation energy. J. Comput. Chem..

[46] Vyazovkin S. (1997). Evaluation of activation energy of thermally stimulated solid-state reactions under arbitrary variation of temperature. J. Comput. Chem..

[47] Vyazovkin S., Dollimore D. (1996). Linear and Nonlinear Procedures in Isoconversional Computations of the Activation Energy of Nonisothermal Reactions in Solids. J. Chem. Inf. Comput. Sci..

[48] Vyazovkin S., Wight C.A. (2000). Estimating Realistic Confidence Intervals for the Activation Energy Determined from Thermoanalytical Measurements. Anal. Chem..

[49] Galukhin A. (2024). Isoconversional approach to quantitative assessment of reactivity under non-isothermal conditions. Thermochim. Acta.

[50] Glasstone S., Laidler K., Eyring H., Hammet L. (1941). The Theory of Rate Processes.

[51] Hein J.E., Fokin V.V. (2010). Copper-catalyzed azide–alkyne cycloaddition (CuAAC) and beyond: New reactivity of copper(i) acetylides. Chem. Soc. Rev..

[52] Galukhin A., Aleshin R., Nosov R., Vyazovkin S. (2023). Probing kinetic and mechanistic features of bulk azide–alkyne cycloaddition. Phys. Chem. Chem. Phys..

[53] Galukhin A., Aleshin R., Nosov R., Vyazovkin S. (2023). Kinetics of Polycycloaddition of Flexible α-Azide-ω-Alkynes Having Different Spacer Length. Polymers.

[54] Vyazovkin S. (2016). A time to search: Finding the meaning of variable activation energy. Phys. Chem. Chem. Phys..

[55] Laidler K. (1981). Symbolism and terminology in chemical kinetics (Appendix no. V to manual of symbols and terminology for physicochemical quantities and units). Pure Appl. Chem..

[56] Rostovtsev V.V., Green L.G., Fokin V.V., Sharpless K.B. (2002). A Stepwise Huisgen Cycloaddition Process: Copper(I)-Catalyzed Regioselective “Ligation” of Azides and Terminal Alkynes. Angew. Chem. Int. Ed..

[57] Muller P. (1994). Glossary of terms used in physical organic chemistry (IUPAC Recommendations 1994). Pure Appl. Chem..

[58] Ruff F., Csizmadia I.G. (1994). Chapter 7—Structure and Reactivity Relationships. Studies in Organic Chemistry.

[59] Fukui K., Yonezawa T., Shingu H. (1952). A Molecular Orbital Theory of Reactivity in Aromatic Hydrocarbons. J. Chem. Phys..

[60] Flachard D., Serghei A., Fumagalli M., Drockenmuller E. (2019). Main-chain poly(1,2,3-triazolium hydroxide)s obtained through AA+BB click polyaddition as anion exchange membranes. Polym. Int..

[61] Vyazovkin S., Sbirrazzuoli N. (2000). Kinetic analysis of isothermal cures performed below the limiting glass transition temperature. Macromol. Rapid Commun..

