# Supplementary material for: Metal-Free A2/B2-Type Azide–Alkyne Polyaddition: Effect of Azides Structure on Their Reactivity and Properties of Polymerization Products
Source: Polymers (Basel). 2025 Jul 10;17(14):1909. doi: 10.3390/polym17141909 (PMC12298694; doi:10.3390/polym17141909)
Supplement: Supplementary file 1 [file polymers-17-01909-s001.zip › polymers-3720316-supplementary.pdf]

Metal-free A_2/B_2-type azide-alkyne polyaddition: effect of azides structure on their reactivity and properties of polymerization products

Andrey Galukhin,[1,*] Roman Aleshin,[1] Alexander Gerasimov,[1] Alexander Klimovitskii,[1] Roman Nosov,[1] Liana Zubaidullina,[1] Sergey Vyazovkin[2,*]

1 *Alexander Butlerov Institute of Chemistry, Kazan Federal University, 18 Kremlevskaya Street, 420008 Kazan, Russian Federation*

2 *Department of Chemistry, University of Alabama at Birmingham, 901 S. 14th Street, Birmingham, AL 35294, USA*

E-mail: and_galuhin@mail.ru, vyazovkin@uab.edu

Supplementary Information

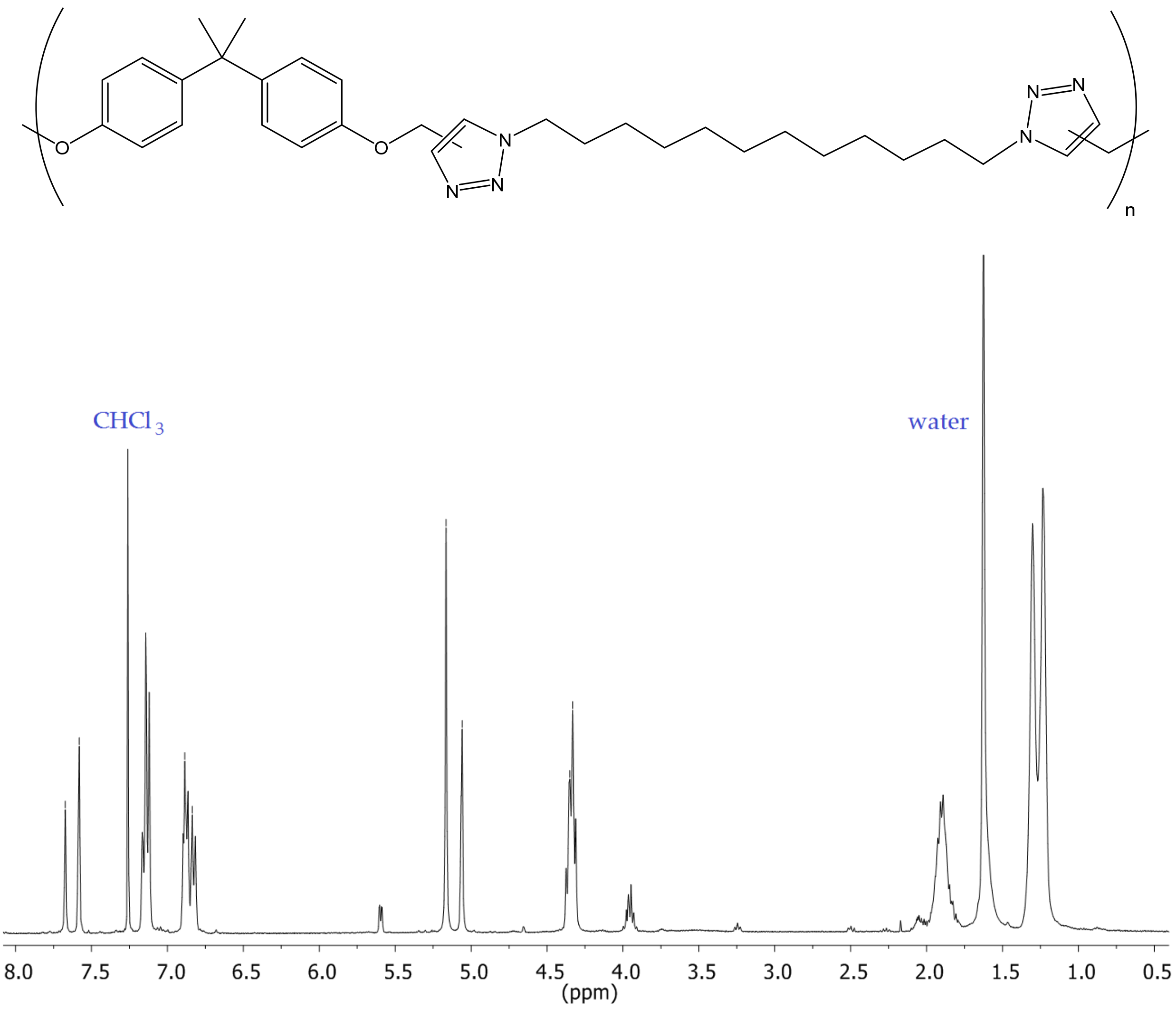

Figure S1. ^{1}H NMR spectrum of polymerization product of dialkyne **1** and diazide **2** (400 MHz, $CHCl_3$-d1, 25 °C).

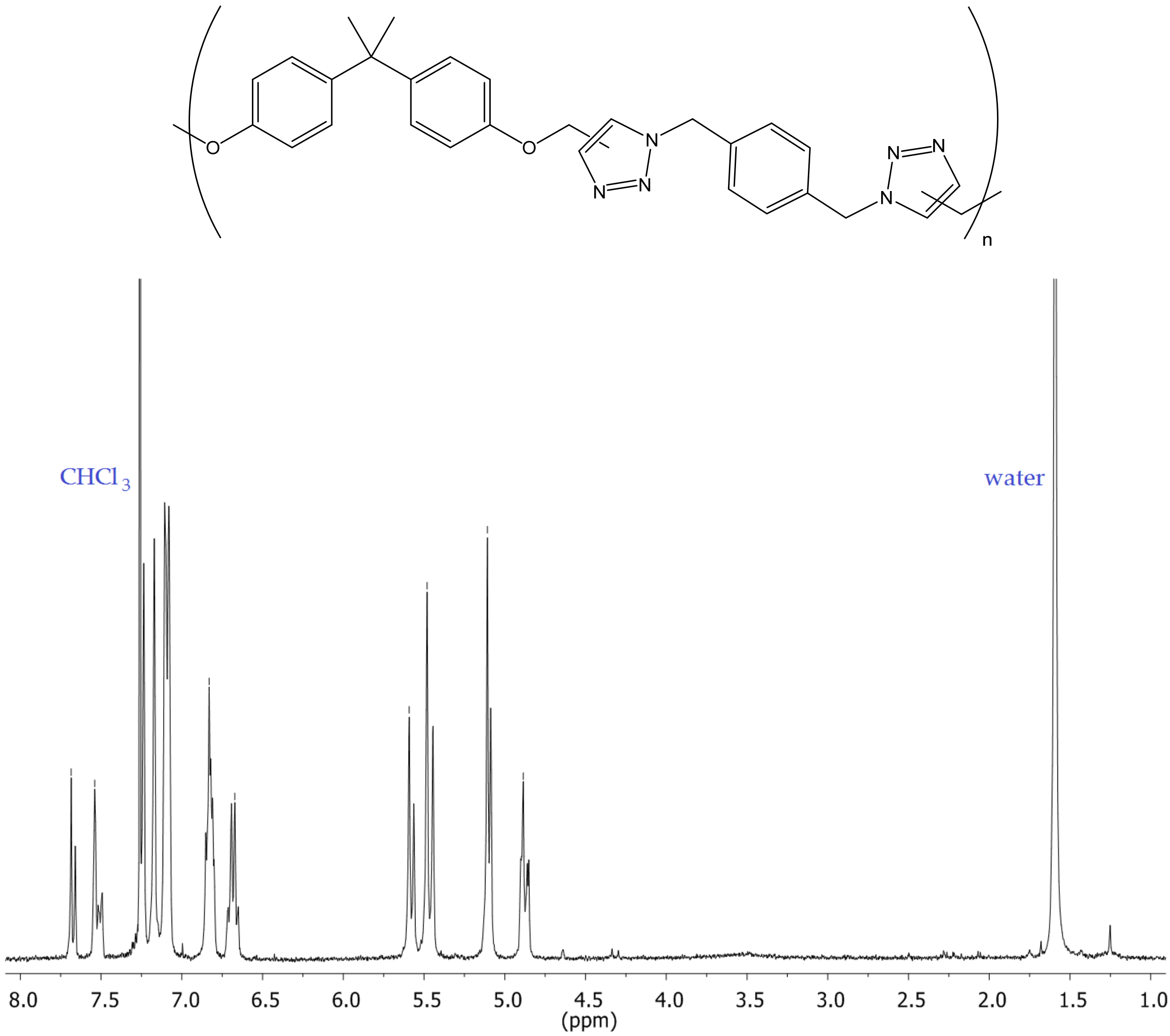

Figure S2. ^{1}H NMR spectrum of polymerization product of dialkyne **1** and diazide **3** (400 MHz, $CHCl_3$-d1, 25 °C).

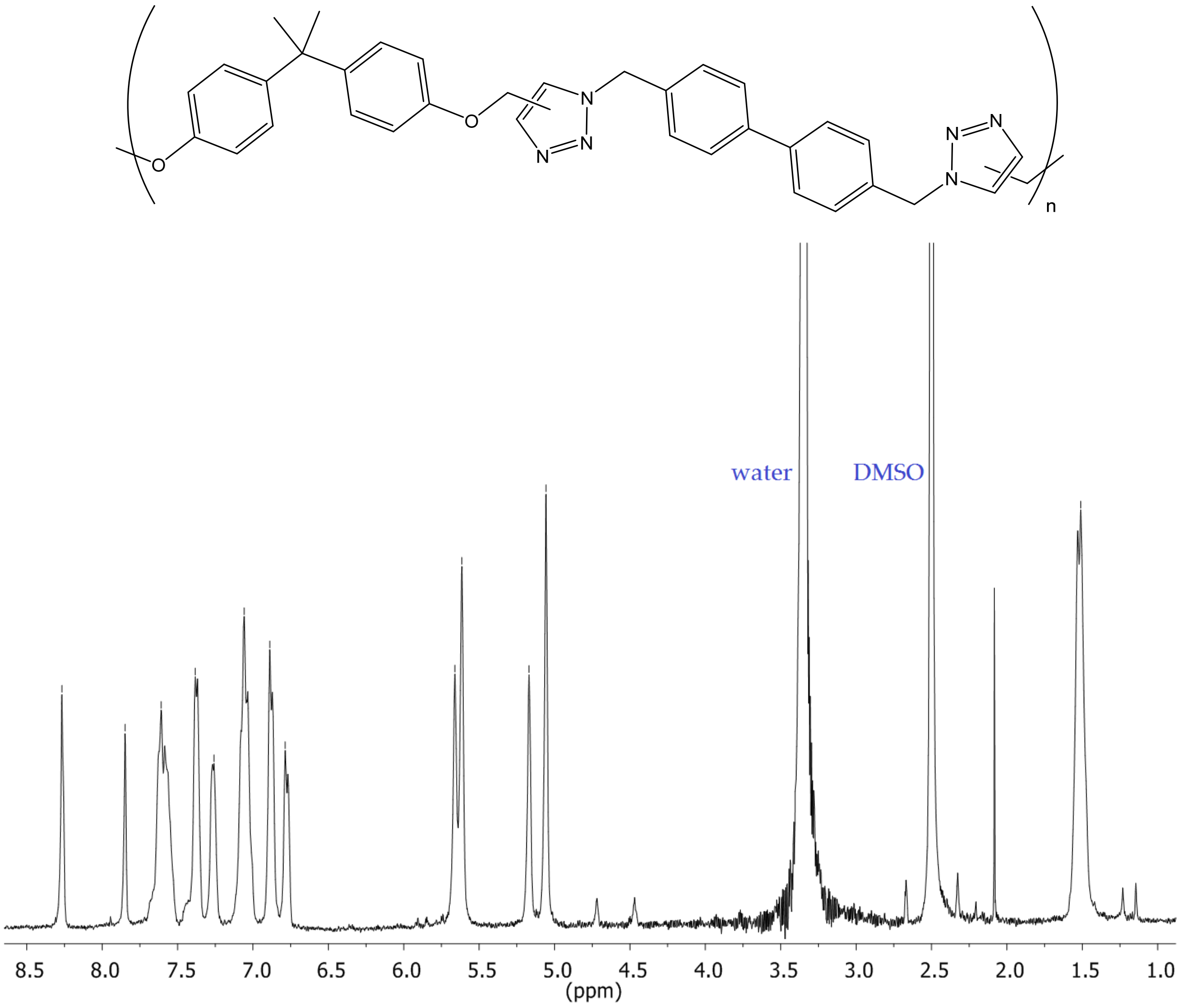

Figure S3. ^{1}H NMR spectrum of polymerization product of dialkyne **1** and diazide **4** (400 MHz, DMSO-d6, 25 °C).
